# Factors influencing high absenteeism rate of student nurses in clinical areas at a nursing college in the Lejweleputswa District

**DOI:** 10.4102/curationis.v42i1.1985

**Published:** 2019-08-26

**Authors:** Griselda N. Magobolo, Barbara M. Dube

**Affiliations:** 1 Free State School of Nursing, Welkom, South Africa; 2 School of Nursing and Public Health, University of KwaZulu-Natal, Durban, South Africa

**Keywords:** absenteeism, clinical areas, student nurses, students’ role, perception

## Abstract

**Background:**

Student nurse attendance during training is mandatory, and the South African Nursing Council (SANC) stipulates that students must attend 80% of hours for both theory and practice during their training. Unauthorised student nurse absenteeism, especially in the clinical areas, has become an increasing problem in nursing education institutions and in the universities. This study explored student absenteeism with the aim of generating solutions that are specifically relevant to the context of the Free State College of Nursing.

**Objectives:**

The objectives of the study were to describe perceived personal reasons and reasons related to clinical areas that contribute to student nurses’ absenteeism from the clinical areas and to explore the relationship between demographic data and reasons for absenteeism.

**Method:**

A quantitative research design with descriptive and exploratory strategies was used. Data were collected by means of a self-administered questionnaire. Data analysis was performed using SPSS version 23.0. One hundred and fifty-two student nurses were sampled after permission to conduct the study was requested and obtained.

**Results:**

The results showed that 72.4% of respondents agreed that students are absent because of physical illness. The majority of students (97.3%) at the selected campus are absent from clinical areas because they are covering staff shortages. The findings showed no particular relationship between gender and absenteeism as absenteeism was present throughout.

**Conclusion:**

Student nurses at a selected campus are generally absent at the clinical areas because they are physically ill and are funded for studying but not paid for working. It was recommended that accurate records of attendance should be kept and absenteeism rates be calculated at frequent intervals.

## Introduction

Student nurse attendance during training is mandatory because it is a crucial requisite in the training and influences the learning outcomes and professionalism (Chukwu et al. 2018:50). Lecturers in nursing colleges and universities are troubled by the high rate of unauthorised student nurse absenteeism because it disrupts the teaching–learning environment and affects the overall well-being of other students (Barlow & Fleischer 2011:228). Absenteeism is an increasing problem and a massive concern in higher education (Olufunmilayo 2017:64; Qutub et al. 2017:1249). Absenteeism inhibits students to attain appropriate information and contact with relevant materials (clinical skills, lectures, practical session) that are required for effective learning to occur (Sharmin et al. 2016:61). Absenteeism is also associated with poor academic performance, unprofessional conduct and inadequate socialisation within the profession (Dashputra et al. 2015:24).

According to the International Council of Nurses Report (ICN 2014:9), insufficient time allocation for students in clinical learning areas hinders the production of clinically safe and competent nursing graduates. Absenteeism in the allocated clinical areas further reduces students’ exposure time and their ultimate level of competency. The Irish Nursing Council (An Bord Altranais 2009:18) stipulates that students must attend 100% of clinical placements each year, and students who have not satisfied this requirement may not progress in the programme. Finland tolerates a 95% attendance to fulfil the requirements of the course, allowing 5% non-attendance. In Nigeria, the University of Abadan authority makes it mandatory for students to attend 75% of lectures before being allowed to sit their examinations (Fayombo, Ogunkola & Olaleye 2012:123).

The South African Nursing Council Regulation 425 (R425) of 1985, as amended, governs qualification and registration as a nurse (general, psychiatry and community) and midwife and provides curriculum guidelines stipulating hours required for both theory and practical exposure of students. According to this regulation, integration of theory and practice requires that student nurses attend a minimum of 80% of both academic and clinical portions of their training programme, thus allowing 20% non-attendance (South African Nursing Council Regulation 425 of 1985).

Because South Africa’s health-care system is predominantly nurse-based and primary health-care-based, it is important that nurses have the necessary expertise to manage the country’s burden of disease. Student nurses’ placement in the clinical areas provides them with opportunities to interact with patients and become socially developed in the practice of nursing (Donnelly & Wiechula 2012:873). Students who are absent from clinical areas miss valuable information on procedures performed in that particular clinical area. According to Dika and Sylejmani (2012:2407), regular attendance in classes and labs correlates directly with students’ success in examinations, a point that is supported by studies on student absenteeism by Andrietti and D’Addazio (2012:10) and Wadesango and Machingambi (2011:93), who reported a relationship between students’ regular attendance and academic achievement.

Free State College of Nursing 2014 statistics indicated high rates of absenteeism among student nurses in both clinical areas and classes, despite regulations to restrain such behaviour. According to the Free State College of Nursing Curriculum Guide for the diploma leading to registration as a nurse (general, psychiatric and community) and a midwife, demotions and termination of training become inevitable should a student fail to attend 80% in these areas. Absenteeism may result in students being barred from examinations because of attendance deficit in either the theoretical or practical components or both. The consequence is that students take longer to complete their training, which has an adverse impact on human resources. Absenteeism not only affects the students themselves but also has financial implications for the Department of Health that is funding their training.

Student absenteeism can be attributed to a number of factors. Thekedam and Kottaram (2015:12) suggest that absenteeism can be linked to lack of interest in the subject matter and poor teaching strategies. Other factors include personal issues such as physical illness, family responsibilities such as a death in the family, and problems with transport to clinical areas (Hidayat et al. 2012:3; Mahmoud 2017:25). According to Aburuz (2015:151), there is very little evidence of policies on student non-attendance in higher education. Support and guidance of students by lecturers and clinical mentors in the clinical areas is very important to boost the confidence of student nurses thus preventing absenteeism. Clinical accompaniment assists the academic staff to ensure that students obtain necessary clinical skills and supervise attendance of students in clinical areas (Van Graan, Williams & Koen 2016:285). However, various studies have proved that there is lack of support from both academic staff and professional nurses in clinical areas that can also contribute to absenteeism of student nurses (Bvumbwe, Malema & Chipeta 2015:929; Rajeswaran 2016:4; Tikawen et al. 2015:69).

Donnelly and Wiechula (2012:875) argue that competent professional nurses require adequate exposure in clinical areas to gain relevant skills and knowledge. However, student nurse absenteeism in clinical areas is a common problem among student nurses of the Free State College of Nursing, irrespective of the monitoring measures that are put in place by nurse educators.

### Purpose of the study

The purpose of the study was to explore and describe factors influencing high absenteeism rate of student nurses in clinical areas at a nursing college in the Lejweleputswa District.

### Objectives of the study

The objectives of the study were:

- To describe perceived personal reasons of student nurses that contribute to absenteeism.
- To describe reasons related to clinical areas that contribute to student nurses’ absenteeism.
- To explore the relationship between demographic data and reasons for absenteeism.

## Methods

A quantitative, descriptive and exploratory design was adopted to explore factors influencing high absenteeism rate of student nurses in clinical areas at a nursing college in the Lejweleputswa District.

### Study respondents and setting

The study was conducted in one of the three campuses of the Free State College of Nursing. The researcher chose this setting because of its accessibility. The population comprised registered student nurses in second, third and fourth year of training who were training for the Diploma in Nursing (General, Psychiatric, Community) and Midwifery. The population size consisted of 76 registered student nurses in second year, 74 in third year and 62 in fourth year of training. The non-probability, convenience sampling method was used to recruit the respondents. This technique was suitable for this study because it was possible that some of the student nurses might be allocated in clinical areas far from Welkom. The researcher chose respondents who were available and ready at the right place and the right time during the study period. The sample size was calculated using a computer program known as Raosoft Sample Calculator, employing the following parameters to ensure representativeness: margin of error of 5% and confidence level of 95%. If 90% of respondents answer yes while 10% answer no, the researcher might be able to tolerate a larger amount of error than if the respondents are split 50–50 or 45–55. The minimum recommended sample size was 163 registered student nurses at a selected nursing college campus in Free State.

### Data collection

The data were collected using a questionnaire that had been adopted from Simelane (2013:161) and Thobakgale (2013:115), related to factors contributing to absenteeism of student nurses. The major concepts in this framework were (1) staff attitudes, (2) shift work, (3) type of unit, (4) mentoring and (5) students’ role. The researcher’s contribution to this study was to describe the five factors believed to influence students’ absenteeism. It was hypothesised that absenteeism of students would be improved if the five factors (staff attitudes, shift work, type of unit, mentoring and students’ role) were properly addressed and monitored.

The researcher obtained ethical clearance from the University of KwaZulu-Natal. She then obtained permission to conduct the study at the selected campus from the Free State Department of Health, the acting Principal of the Free State College of Nursing and the Dean of the selected campus. The validity of the questionnaire was maintained by ensuring that it contained the same questions for all respondents. The validity of this study was determined through content validity and face validity. The researcher checked the items in the data collection instrument against the research objectives and conceptual framework to determine whether they measured all of the elements of interest in the study. The reliability of the questionnaire was measured by conducting a pilot study with six respondents to determine its stability and consistency.

The instrument was administered a week before data collection to six respondents from the sample who did not form part of the study. As soon as the respondents agreed to participate in the study, they were requested to sign consent forms. The researcher explained to them how to complete the questionnaire to avoid errors, wastage and spoiled questionnaires. Thereafter, the questionnaires were distributed by the researcher herself to the students in the classroom at the selected campus over a period of 3 weeks to a group of 163 students. Only 152 (93%) returned properly completed questionnaires which, according to Grove et al. (2013:355), is an acceptable response rate.

### Analysis

The primary source used for data collection was a questionnaire. Data were generated, organised and analysed using the Statistical Package for Social Science (SSPS), Version 23.0, with the help of the University KwaZulu-Natal statistical consultation service. Descriptive statistics were used to calculate the mean and mode. The chi-square test was also considered to compare reasons from the clinical areas that might contribute to student absenteeism.

### Ethical considerations

Ethical approval for this study was obtained from the College of Health Sciences of the University of KwaZulu-Natal Research Ethics Committee with ethical clearance number HSS/1441/015M. When this was available, permission to proceed with the study was granted by the Acting Principal of the Free State School of Nursing and the Dean of the selected campus. The respondents were given information sheets, after which informed consent forms were signed and students were assured of their privacy, confidentiality and anonymity.

## Results

A total of 163 questionnaires were handed out to the respondents of which 152 were returned, giving a 93% response rate. The minimum age of the respondents was 19 years, and the maximum age was 46 years. The majority of respondents 121 (79.6%) were females compared to 31 (20.4%) males. The majority of the student nurses, 121 (79.6%), lived in the nurse’s residence; 29 (19.1%) lived at home and 2 (1. 3%) rented elsewhere. Most respondents 64 (42.1%) were in the second year of study, followed by 50 (32.9%) in third year and 38 (25.0%) in fourth year.

On personal reasons for absenteeism, the majority of respondents, 145 (88.5%), agreed that they are absent because they attend to family challenges, for example sick child, spouse or parent. Physical illness was also perceived by the majority of respondents 110 (72.4%) as a reason for absenteeism. Furthermore, 93 (57.0%) agreed that students are absent because they are funded for studying but not being paid for working.

The majority of respondents, 136 (89.4%), agreed with the reason that students are absent in the clinical areas because of work overload; 126 (77.3%) respondents agreed with the reason that students are absent from the clinical area because they are ill-treated by senior staff. Furthermore, 115 (70.5%) respondents agreed that students are absent because they do not want to be treated as workforce. Regarding the reason students are absent because they are avoiding certain shifts, 114 (69.9%) respondents agreed. On the reason that students are absent because they are not given days off that they requested, 104 (63.8%) respondents agreed.

The Pearson’s chi-square test for clinical area reasons contributing to absenteeism indicated that there is no statistical difference between age and the reason that students cover staff shortages (*p*-value 0.250), or between age and the reason that students are ill-treated by senior staff (*p*-value 0.121). Thus, students generally agreed with the above reasons irrespective of their age. Furthermore, with regard to age and the reason for student absence being to avoid certain wards with very sick patients, the Pearson’s chi-square test showed no significant difference (*p*-value 0.598); a majority of respondents in the age range 18–30 years agreed.

The Pearson’s chi-square test for clinical area reasons contributing to absenteeism indicated that there is no statistical difference between gender and the reason that there is work overload (*p*-value 0.211), between gender and the reason that the students are ill-treated by senior staff (*p*-value 0.360), or between gender and avoidance of certain shifts (*p*-value 0.229). Thus, students generally agreed with the above reasons irrespective of their gender.

The Pearson’s chi-square test for clinical area reasons contributing to absenteeism indicated that there is no statistical difference between year of training and the reason that there is work overload (*p*-value 0.502), between year of training and the reason that the students are ill-treated by senior staff (*p*-value 0.981), or between year of training and avoidance of certain shifts (*p*-value 0.329). Thus, students generally agreed with the above reasons irrespective of their year of training.

## Discussion of results

The study was guided by the objectives, and a quantitative research design with descriptive and explorative strategies was used. In this section, demographic profile of the respondents, personal circumstances of the students contributing to absenteeism as well as reasons from the clinical areas perceived by students as contributing to absenteeism will be reported.

### Demographic profile of respondents

On the suggestion that avoiding certain wards with very sick patients was a reason for students’ absence from clinical areas, 54% of respondents in the age range 18–30 years agreed, while 52.4% of those in the age range 31–50 years disagreed (*p*-value 0.598). These findings correspond with those by Simelane (2013:143) which showed that younger students were more likely to be absent than the older ones. However, a study by Desalegn et al. (2014:6) found, on the contrary, that older students were more likely to be absent than the young ones. Singh (2012:55) found no significant relationship between age and absenteeism. This study found no particular relationship between gender and absenteeism, as absenteeism was present throughout. Deane and Murphy (2013:2287) and Wadesango and Machingambi (2011:91) found that male students were more frequently absent than their female counterparts. Desalegn et al. (2014:6) and Simelane (2013:144), on the other hand, found no relationship between absenteeism and gender. In this study, there was no particular relationship between residence and absenteeism, as absenteeism was present throughout. However, Bati et al. (2013:591) found that students living closer to campus reported fewer absences than those living further away.

### Personal circumstances of the students contributing to absenteeism

The results of this study showed that 95.4% of respondents agreed that students might be absent because of family challenges they have to attend. This corresponds with the finding by Bradshaw and Lowenstein (2014:35) that family reasons such as looking after a sick relative and funeral are excuses for student absenteeism.

The findings in this study are similar to those in the study by Sarkodie et al. (2014:18), who found that physical illness is a cause of absenteeism among students. In this study, the majority of respondents, 72.4%, agreed that students may be absent because of physical illness. The findings also showed that a majority of student nurses, 61.2%, agreed, while 38.8% disagreed that they are generally absent because they are funded for studying but not paid for working. These findings are contrary to those by Simelane (2013:145) which reflected that students’ stipend does not contribute to absenteeism of bursary students.

### Clinical area reasons perceived by students as contributing to absenteeism

On the point that students may be absent from the clinical areas because they are covering staff shortages, 97.3% of the respondents agreed. This corresponds with the finding by Msiska, Smith and Fawcett (2014:38) that students on clinical areas cover staff shortages and are regarded as additional staff. In the findings in this study on work overload as a reason for student absence, 89.4% of the respondents agreed. This corresponds with the finding by Abdelrahman and Abdelkade (2017:67) that one of the reasons why students were absent in the clinical areas was work overload. In the study by Mothobi (2017:82), students indicated that staff members were inhuman towards them, thus encouraging absenteeism. The majority of the respondents in this study, 82.9%, agreed that students may be absent because they are ill-treated by senior staff. In the study by Mathebula (2016:96), students revealed that they are blamed for everything bad that is happening in the clinical areas.

In this study, 75.7% agreed that students may be absent because they do not want to be treated as workforce. This corresponds with the findings by Van Graan et al. (2016:286) and Singh (2015:56) that students may be absent in the clinical areas because they do not want to be treated as workforce. In this study, 75% of respondents agreed that students may be absent because they avoid certain shifts. This corresponds with the finding by De Lima et al. (2016:3433) that students may be absent in the clinical areas because they avoided 12-h shifts.

Regarding the suggestion that students may be absent because they are not given days off that they requested, 68.4% of respondents agreed. This corresponds with the finding by Simelane (2013:152) that students were absent in the clinical areas because they were not given days off that they had requested. However, a study by Singh (2012:43) found no significant relationship between student absenteeism and not being given the days off they requested.

## Recommendations

Student absenteeism interferes with the learner’s progress and is costly to the state because these students are subsidised for their studies. Based on the study results and literature review, the researcher puts forward the following recommendations to curb absenteeism. These recommendations include suggestions for nursing practice, nursing education and nursing research.

### Nursing practice

- In-service training of all permanent employees with regard to their attitudes towards student nurses in the clinical areas.
- Proper communication in the form of regular meetings between qualified staff members, lecturers and students to identify problems experienced by students in the clinical areas, thereby improving working conditions of the students.

### Nursing education

- Attendance policies that include best practices should be developed, consistently enforced and attendance policy evaluation conducted.
- Accurate records of attendance should be kept and absenteeism rates should be calculated at frequent intervals to identify the pattern of absenteeism for each student.
- Supervision and support of students at the clinical areas by academic staff to address concerns of students.

### Nursing research

- Additional study is recommended on the same phenomenon that would include all the campuses of the Free State College of Nursing.
- The same absenteeism phenomenon should be investigated as perceived by nurse educators and clinical supervisors.

## Limitations of the study

The researcher selected one campus of the three in the Free State College of Nursing because of limited funding, but hopes that the findings may also be applicable to other campuses because they share common characteristics.

## Conclusion

The findings of the research indicated that when student nurses at the selected campus were absent at clinical areas, this was generally because they are physically ill, attend to family problems and are funded for studying but not paid for working. The findings showed that younger students ranging between 18 and 30 years were absent because they avoid certain wards with sick patients. In addition, there was no particular relationship between gender and absenteeism.

Empirical evidence has shown that students may be absent in the clinical area because of work overload, because they cover staff shortages and because they are ill-treated by senior staff. Student nurse absenteeism was thus evident in the clinical area.
